# Gut bacterial communities across 12 Ensifera (Orthoptera) at different feeding habits and its prediction for the insect with contrasting feeding habits

**DOI:** 10.1371/journal.pone.0250675

**Published:** 2021-04-26

**Authors:** Xiang Zheng, Qidi Zhu, Zhijun Zhou, Fangtong Wu, Lixuan Chen, Qianrong Cao, Fuming Shi

**Affiliations:** 1 College of Life Science, Institute of Life Science and Green Development, Hebei University, Baoding, China; 2 Laboratory of Enzyme Preparation, Hebei Research Institute of Microbiology, Baoding, China; Indian Agricultural Research Institute, INDIA

## Abstract

Insect microbial symbioses play a critical role in insect lifecycle, and insect gut microbiome could be influenced by many factors. Studies have shown that host diet and taxonomy have a strong influence on insect gut microbial community. In this study, we performed sequencing of V3-V4 region of 16S rRNA gene to compare the composition and diversity of 12 Ensifera from 6 provinces of China. Moreover, the influences of feeding habits and taxonomic status of insects on their gut bacterial community were evaluated, which might provide reference for further application research. The results showed that Proteobacteria (45.66%), Firmicutes (34.25%) and Cyanobacteria (7.7%) were the predominant bacterial phyla in Ensifera. Moreover, the gut bacterial community composition of samples with different feeding habits was significantly different, which was irrespective of their taxa. The highest diversity of gut bacteria was found in the omnivorous Ensifera. Furthermore, common and unique bacteria with biomarkers were found based on the dietary characteristics of the samples. However, the bacterial community structure of the Ensifera samples was significantly different from that of Caelifera. Therefore, we concluded that feeding habits and taxonomic status jointly affect the gut bacterial community composition of the samples from Orthoptera. However, the influence of feeding habit dominates when taxonomy category below the suborder level. In addition, the dominant, common and unique bacterial community structure could be used to predict the contrastic feeding habits of insects belonging to Ensifera.

## Introduction

Insects, with abundant species, diverse ecological habits and large-scale biomass, make an important contribution to the world’s ecosystems. Insect microbial symbionts, especially beneficial microorganisms, play an important role in the diversity and evolution of insects. Moreover, many beneficial microbes are found mainly in insect intestinal system [1,2]. Gut microbes may contribute to insects by providing essential nutrition with amino acids, vitamins and nitrogen, detoxifying plant defence compounds, enhancing pathogen resistance, influencing social interactions and affecting growth, development, reproduction and longevity [3–6] and more.

Gut microbial community structure is influenced by many factors such as insect phylogeny, habitat, life stage, sex, feeding habits [7,8]. Notably, scientific evidence has shown that host diet as an exogenous factor and host phylogeny as an endogenous factor strongly affect microbial community structure [9–11]. Meanwhile, due to the remarkable difference in diet types, some previous studies have reported that host diet is the primary determinant in altering the gut bacterial community structure, such as *D*. *melanogaster* [12], higher termites [13], *Dastarcus helophoroides* [14], wild dragonflies [15], bee [9] and beetles [16,17]. However, other studies have shown that the gut bacterial community proportion remains stable compared with dietary alteration, suggesting that host phylogeny as an endogenous factor greatly affects the gut bacterial community structure, such as cricket [18], butterflies [19] and cockroach [20,21].

China is one of the richest countries with Ensifera record in the world. So far, 677 species (subspecies) belonging to 149 genera of 11 subfamilies which performed highly species diversity and diversified feeding habits have been recorded [22]. They are important pests in agriculture and forestry, as well as potential resources in biological control and utilization of insect natural enemies.

It is essential to understand the relationships between Ensifera and its gut bacterial community for better usage of the potential role of gut microbes based on the two most important factors: taxonomy and feeding habits. Notably, many studies have mostly focused on altering host diet to observe changes in its gut microbes, and wild insect samples have been found to better reflect the microbial community structure contained in the gut [18,23]. However, reports on these species are very limited. In this study, the gut bacterial community compositions from 12 Ensifera associated with different feeding habits have being investigated by sequencing the V3-V4 region of the 16S rRNA gene. Additionally, this study aimed to get new knowledge on whether some regular features shown by the gut bacterial community are dependent on their host diet or phylogeny or both.

## Material and methods

### Sample collection and DNA extraction

The samples were collected using sweep nets at eight wild sites based on prior diet records and natural history on their habitat in six provinces across China in 2019 (Table 1) [22]. During the process of collection, we focused on the insect samples of different feeding habits and belonged to different families. The Department of Forestry of Guangxi Zhuang Autonomous Region approved us to enter Daming Mountains, Dayao Mountains and Huaping (Longsheng county) National Nature Reserve to collect insect samples. Zhejiang Tianmu Mountains National Nature Reserve Administration approved us to enter the Tianmu Mountains National Nature Reserve to collect insect samples. Hubei Shennongjia National Nature Reserve Administration approved us to enter the Xinhua forestry station to collect insect samples. Chongqing Simian Mountain Nature Reserve Management Committee approved us to enter Simian Mountain Nature Reserve to collect insect samples. Samples from Hebei and Henan provinces were collected in farmland. All samples were adults and preserved in 99% (v/v) ethanol immediately after capture until identification and dissection [1]. Samples were identified and recorded based on morphological characteristics.

**Table 1:** Sampling information.

Taxonomy			Feeding habits	Location		Number of samples	Abbreviations
Suborder	Family	Species		County/Mountain, Province	Geographic coordinates		
Ensifera	Tettigoniidae	*Gampsocleis gratiosa*	Equivocal	Yichuan county, Henan	112°30′ E 34°31′ N	6	Gam
		*Hexacentrus japonicus*	Carnivores	Tianmu mountains, Zhejiang	119°43′ E 30°35′ N	3	Hex
		*Tegra novaehollandiae viridinotata*	Herbivores	Simian mountains, Chongqing	106°39′ E 28°62′ N	3	Teg
		*Phyllomimus sinicus*	Herbivores	Daming mountains, Guangxi	108°34′ E 23°52′ N	5	Phy
		*Mecopoda niponensis*	Herbivores	Tianmu mountains, Zhejiang	119°43′ E 30°35′ N	5	Mec
	Gryllacrididae	*Ocellarnaca emeiensis*	Carnivores	Daming mountains, Guangxi	108°34′ E 23°52′ N	5	Oce
		*Capnogryllacris spinosa*	Carnivores	Longsheng county, Guangxi	109°94′ E 25°60′ N	4	Cap
	Rhaphidophoridae	*Diestramima excavata*	Omnivores	Dayao mountains, Guangxi	110°11′ E 24°14′ N	5	Die_e
		*Diestramima Beybienkoi*	Omnivores	Shennongjia mountains, Hubei	110°89′ E 31°60′ N	5	Die_b
		*Rhaphidophora incilis*	Omnivores	Daming mountains, Guangxi	108°34′ E 23°52′ N	5	Rha
	Gryllotalpidae	*Gryllotalpa orientalis*	Equivocal	Quyang county, Hebei	114°78′ E 38°59′ N	5	Gry
	Gryllidae	*Duolandrevus dendrophilus*	Equivocal	Daming mountains, Guangxi	108°34′ E 23°52′ N	5	Duo
Caelifera	Acrididae	*Acrida cinerea*	Herbivores	Quyang county, Hebei	114°78′ E 38°59′ N	6	Acr

Each sample was gently dissected with fine-tipped forceps in a clean Petri dish under sterilized condition, and was performed by collecting the midgut and hindgut with gut contents [18,24,25]. Gut sections from each sample were processed to extract DNA following TIANamp Stool DNA Kit (TIANGEN, China) according to the manufacturer’s protocols [26,27].

To reduce the chance of cross-contamination, dissection of each sample started with 70% ethanol solution, rinsed and wiped [28]. Following the extraction, the total DNA in each sample was measured using a NanoDrop 2000 spectrophotometer (Thermo Fisher Scientific, USA). The total extracted DNA was stored at −20°C until it was sequenced.

### PCR amplification and 16S rRNA sequencing

Genomic DNA extracted from the samples was amplified using the V3–V4 hypervariable regions of the bacterial 16S rRNA gene. The primers were as follows: the forward 341F (5′-CCTACGGGNG GCWGCAG-3′) and reverse 805R (5′-GGACTACHVGGGTWTCTAAT-3′) [9,29,30]. PCR amplification was performed using 25 μL reaction mixture containing 12.5 μL PCR Premix, 2.5 μL of each primer, 25-ng template DNA and PCR-grade water to adjust the volume. The PCR conditions profile was as follows: initial denaturation at 98°C for 30 s, followed by 32 cycles of denaturation at 98°C for 10 s, annealing at 54°C for 30 s, extension at 72°C for 45 s and then final extension at 72°C for 10 min. The PCR products were examined by 2% agarose gel electrophoresis, purified by AMPure XT beads (Beckman, USA) and quantified by Qubit (Invitrogen, USA). Finally, the amplicon pools were prepared for sequencing and the size and quantity of the amplicon library were assessed on an Agilent 2100 Bioanalyzer (Agilent, USA) and using the Library Quantification Kit for Illumina (Kapa Biosciences, USA), respectively. The samples were sequenced on an Illumina NovaSeq platform according to the manufacturer’s recommendations provided by LC-Bio, China [31].

### Data and bioinformatics analysis

Paired-end reads was assigned to samples based on their unique barcode and truncated by cutting off the barcode and primer sequence. Paired-end reads were merged using FLASH. Quality filtering on the raw reads were performed under specific filtering conditions to obtain the high-quality clean tags according to the fqtrim(v0.94). Chimeric sequences were filtered using Vsearch software(v2.3.4) [32]. After dereplication using DADA2, which generated a parametric error model and trained on all raw sequencing data and used the model to correct and collapse sequencing errors into ASVs, we obtained feature table and feature sequence [33].

Alpha diversity and beta diversity were calculated by normalising the same sequences randomly. Then, according to SILVA (release132) classifier, feature abundance was normalised using relative abundance of each sample. Alpha diversity was used for analysing complexity of species diversity for a sample through 5 indices, including Chao1, Observed species, Goods coverage, Shannon and Simpson, all these indices in our samples were calculated with QIIME2. Beta diversity was calculated using QIIME2, and determined using principal component analysis (PCA) and principal coordinates analysis (PCoA) according to the unweighted unifrac distance metrics for bacteria to evaluate the similarity between samples, the graphs were drawn using R package. Blast was used for sequence alignment, and the feature sequences were annotated using SILVA database for each representative sequence. A clustered analysis with the corresponding dendrogram of the gut bacterial community was determined and displayed in a heat map. Other diagrams were implemented using the R package (v 3.5.2). Analysis of similarity (ANOSIM) was used to determine the differences between samples, and *p* < 0.05 was considered statistically significant difference [29,34].

Additionally, a linear discriminant analysis effect size (LEfSe) was performed to identify biomarkers associated with samples of the three feeding habit groups using bacterial features [35]. Bacterial abundance profiles were calculated at taxonomic levels of phylum and genus and a logarithmic LDA score ≥ 3.0 was used as thresholds. Based on the occurrence rules of biomarkers, common and unique bacteria of the samples, the insects with contrasting feeding habits have been analysed, and then predicted.

## Results

The gut bacterial communities of 62 specimens representing 13 species (12 species of Ensifera and 1 species of Caelifera) of Orthoptera were compared and analyzed. According to taxonomical identification, these adult samples were identified as species of *Gampsocleis gratiosa* (n = 6), *Hexacentrus japonicus* (n = 3), *Tegra novaehollandiae viridinotata* (n = 3), *Phyllomimus sinicus* (n = 5), *Mecopoda niponensis* (n = 5), *Ocellarnaca emeiensis* (n = 5), *Capnogryllacris spinosa* (n = 4), *Diestramima excavata* (n = 5), *Diestramima Beybienkoi* (n = 5), *Rhaphidophora incilis* (n = 5), *Gryllotalpa orientalis* (n = 5), *Duolandrevus dendrophilus* (n = 5) and *Acrida cinerea* (n = 6), and classified based on carnivores, omnivores, herbivores and equivocal feeding habits from six family taxa. Meanwhile, the samples from the Tettigoniidae family comprised varying feeding habits. However, samples from the families Gryllacrididae and Rhaphidophoridae comprised a single type of feeding habit, and feeding habits of *G*. *gratiosa*, *G*. *orientalis* and *D*. *dendrophilus* were contrast, whose feeding habits were defined as equivocal (Table 1). Abbreviations are given in the table according to the scientific names of Ensifera.

### Gut bacterial community diversity

**Fig 1:**
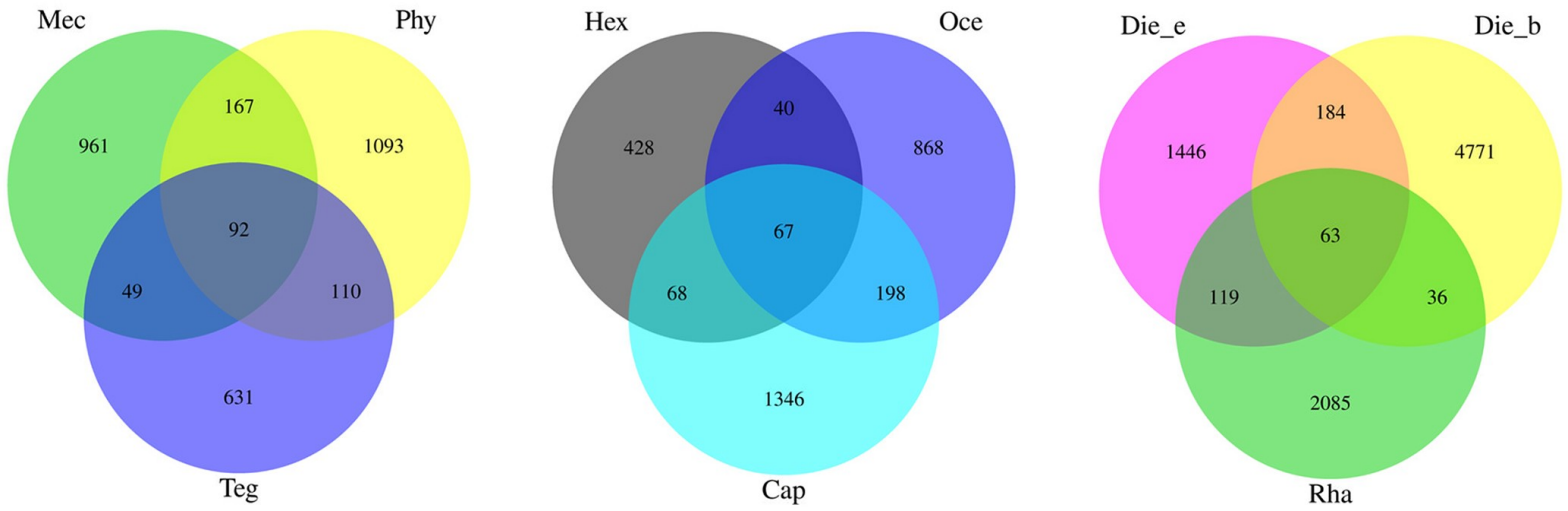
Venn diagram of the gut bacterial communities features in different feeding habits. Shared features were shown in core.

After quality filtering and dereplication by QIIME2, feature sequences from the gut samples of 13 insect species were successfully obtained. Rarefaction indices suggested that sampling depth was sufficient to determine the bacterial community composition [36]. Venn diagram analysis showed that 4 features were shared among these samples. Overall, 5049 features were found in *D*. *beybienkoi*, which harboured the highest feature number among all samples, whereas *G*. *gratiosa* harboured the lowest feature number of 475. The mean features of all species were 1761.23 (S1 Fig). Furthermore, herbivores, carnivores and omnivores shared 92, 67 and 63 features, constituting approximately 2.96%, 2.22% and 0.72% of all features, respectively (Fig 1). While the total number of gut bacterial features in omnivores was highest, the percentage of common features was below that of herbivores and carnivores, indicated that omnivorous insects have a higher gut bacterial community diversity.

Alpha diversity indices indicated that there were significant differences in the gut bacterial communities among all insect samples (Kruskal–wallis, *p* < 0.05). The gut bacterial communities from *D*. *beybienkoi* showed the highest bacterial richness values (Observed_species: 1156.20 ± 627.67, Chao1:1183.90 ± 644.15) and *G*. *orientalis* exhibited the highest bacterial diversity indices (Shannon: 6.53 ± 0.72, Simpson: 0.95 ± 0.05) (Table 2). In general, the diversity and richness of gut bacterial communities of omnivorous insects were higher than those herbivorous and carnivorous insects, suggested that the diversity of gut bacterial of insects fed on complex diets was higher (S2 Fig).

**Table 2:** Alpha diversity analysis of the gut bacterial communities.

Abbreviations	Richness estimates		Diversity estimates	
	Observed_ species	Chao1	Shannon	Simpson
**Herbivores**				
Mec	317.60 ± 28.75	324.08 ± 150.68	4.19 ± 1.25	0.80 ± 0.16
Phy	387.60 ± 250.59	395.06 ± 254.68	3.46 ± 1.89	0.66 ± 0.24
Teg	423.67 ± 118.11	326.11 ± 119.06	4.64 ± 0.53	0.83 ± 0.12
**Carnivores**				
Hex	222.33 ± 28.75	229.68 ± 30.39	2.73 ± 1.00	0.68 ± 0.12
Cap	472 ± 296.12	484.93 ± 297.40	2.86 ± 0.71	0.65 ± 0.15
Oce	295.40 ± 64.99	303.46 ± 64.31	3.62 ± 1.41	0.78 ± 0.22
**Omnivores**				
Die_b	1156.20 ± 627.67	1183.90 ± 644.15	5.07 ± 1.82	0.78 ± 0.15
Die_e	440.20 ± 266.36	452.13 ± 271.87	3.08 ± 1.01	0.66 ± 0.17
Rha	558.80 ± 191.31	570.05 ± 193.20	5.30 ± 1.05	0.89 ± 0.07
**Equivocal**				
Duo	742.40 ± 269.59	757.76 ± 276.85	5.82 ± 1.21	0.89 ± 0.09
Gry	712 ± 136.98	721.36 ± 141.71	6.53 ± 0.72	0.95 ± 0.05
Gam	131.17 ± 34.87	136.39 ± 36.10	2.94 ± 0.78	0.73 ± 0.11
**Sample from Caelifera**				
Acr	179 ± 77.88	179.99 ± 78.19	2.86 ± 0.71	0.65 ± 0.15
Kruskal–wallis	p = 0.00031	p = 0.00036	p = 0.00077	p = 0.0089

Values represent the Kruskal–wallis (*p*-value) for each term. Terms (*p* < 0.05) indicated statistically significant.

**Fig 2:**
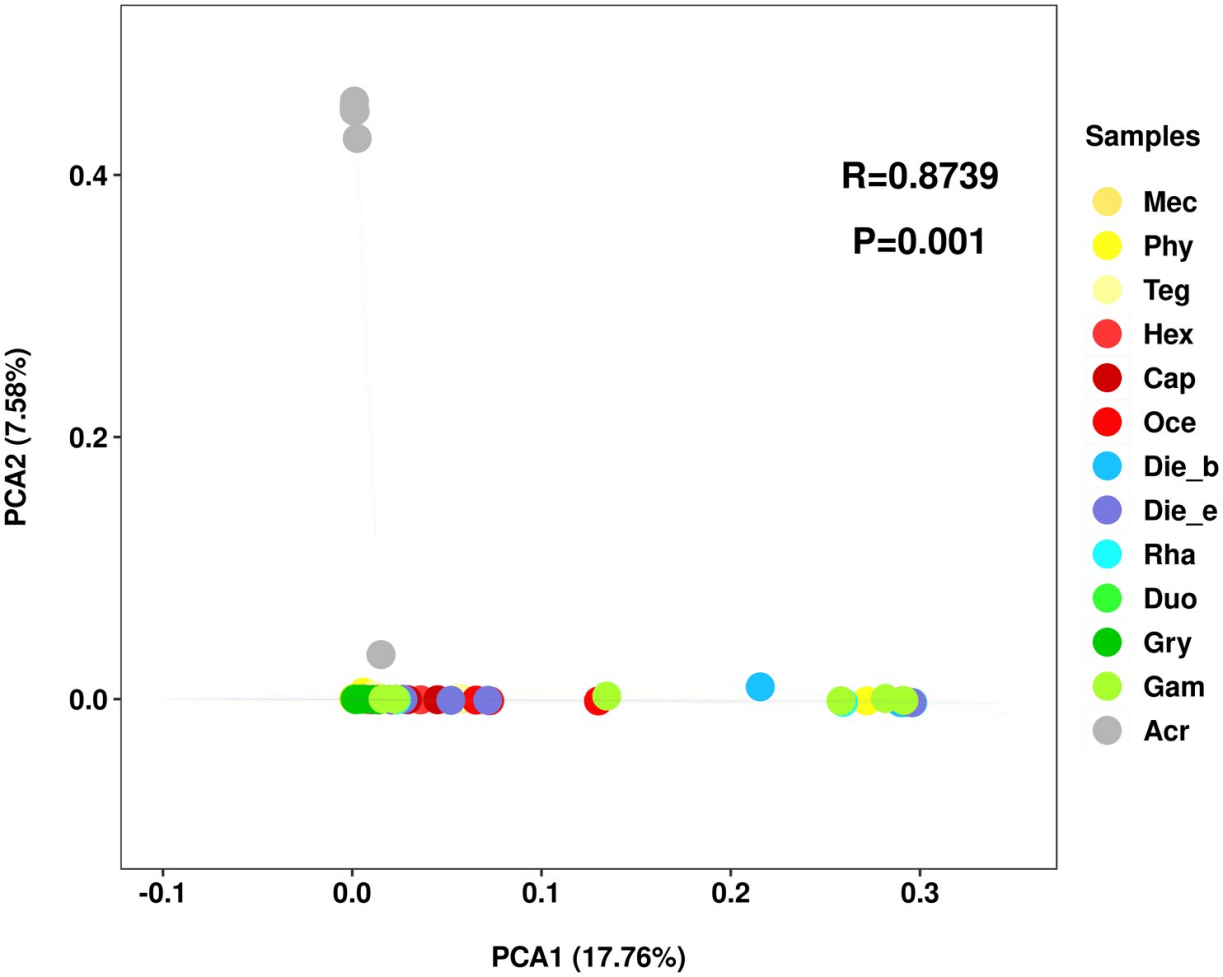
Bacterial communities clustered by using principal component analysis (PCA).

Based on the abundance of features, PCA was used to show the clustered gut bacterial communities. Observably, the gut bacterial community composition of *A*. *cinerea* (Caelifera) clustered separately far away from the insect samples belonged to Ensifera, whereas insect samples belonged to Ensifera were clustered closely (Fig 2). This analysis indicated that the bacterial communities were influenced by taxonomic status more than feeding habits at suborder level of insect samples, but showed slight differences at family level (ANOSIM, R = 0.8739, *p* = 0.001).

**Fig 3:**
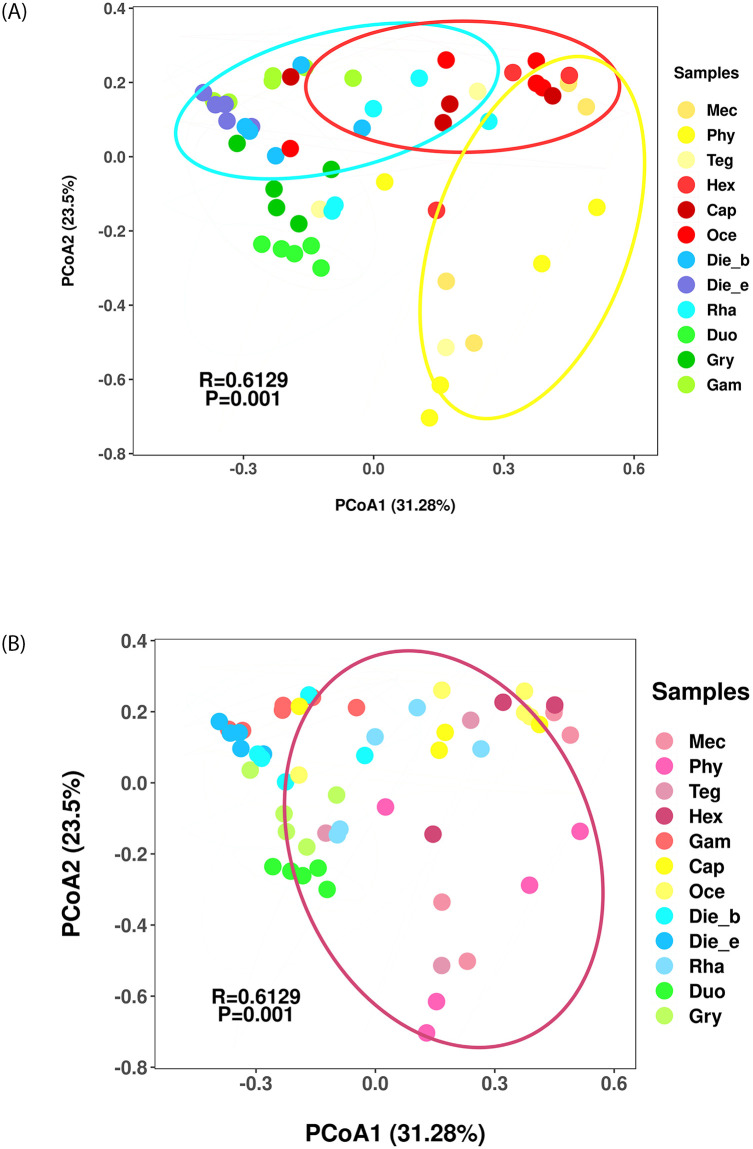
Bacterial communities clustered by using principal coordinates analysis (PCoA) based on the weighted unifrac distance. Eigenvalues of PCoA1 and PCoA2 are shown in parentheses. Samples were colored according to insects feeding habits (A) and insects taxonomy on family level (B).

Similarly, the PCoA revealed similarities in the gut bacterial communities among insects from Ensifera, indicating that gut bacterial communities clustered by feeding habits and taxonomic status of insects contributed significantly to the bacterial community composition (ANOSIM, R = 0.6129, *p* = 0.001). However, feeding habits may contribute more than taxonomic status as driving forces for the observed bacterial community differences. Insects with different feeding habits showed a clear separation in the bacterial communities, although the plots overlap among samples (Fig 3A). In contrast, the bacterial communities did not cluster closely by insect taxonomy at family level, especially insects from the Tettigoniidae family contained varying feeding habits, which overlapped with the samples from families of Gryllacrididae and Rhaphidophoridae (Fig 3B). Overall, although feeding habits and taxonomic status were important factors influencing variation of insect samples from Ensifera, feeding habits account for a much larger proportion of variation than taxonomic status did.

### Gut bacterial community composition

**Fig 4:**
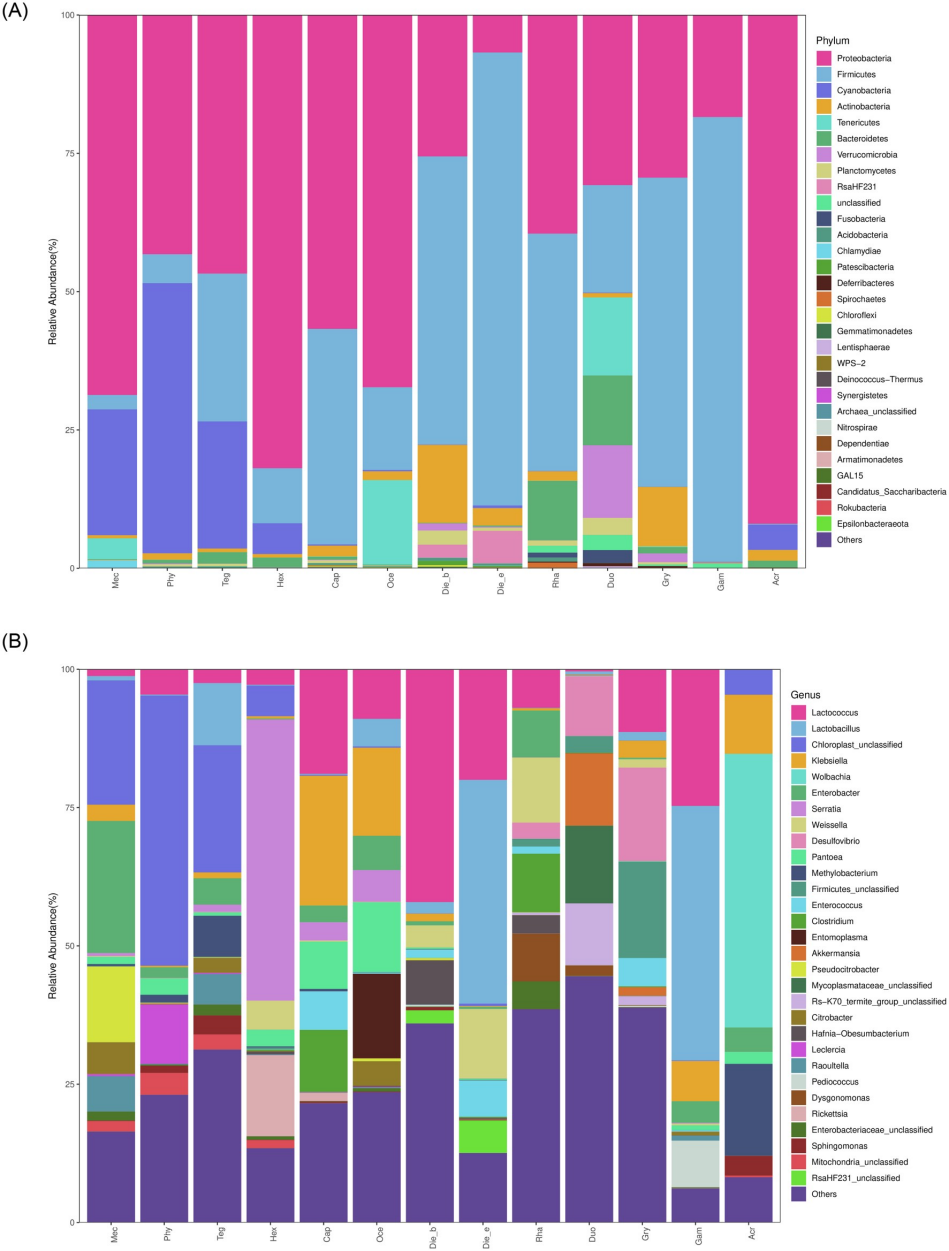
Bacterial community composition across samples at (A) the phylum and (B) the genus levels. Taxa without a top 30 abundant are indicated as others.

Bacterial communities of 41 bacterial phyla represented 120 classes, 272 orders, 504 families and 1284 genera have been detected in the samples. At phylum level, a comparison analysis of bacterial community structures revealed that Proteobacteria, Firmicutes, Cyanobacteria, Actinobacteria, Tenericutes and Bacteroidetes were the predominant phyla, whereas Proteobacteria, Firmicutes and Cyanobacteria with a mean abundance of 45.66%, 34.25% and 7.70%, respectively. Proteobacteria were the largest components in herbivorous and carnivorous insects, especially in *H*. *japonicas* with the relative abundance was 81.91%. Firmicutes were highly dominant in omnivorous insects with the highest relative abundance of 81.88% in *D*. *excavate* (Fig 4A, Table 3). It was found that the species abundance of gut bacteria in *A*. *cinerea* was low, more than 5% relative abundance bacterial phylum was Proteobacteria of 91.94%.

**Table 3:** Top 4 abundance gut bacteria at the phyla and genera levels of samples with different feeding habits.

Herbivores				Carnivores				Omnivores			
Phyla/%		Genera/%		Phyla/%		Genera/%		Phyla/%		Genera/%	
Proteobacteria	52.88%	Chloroplast_unclassified	31.42%	Proteobacteria	68.63%	*Serratia*	19.95%	Firmicutes	58.95%	*Lactococcus*	22.99%
Cyanobacteria	31.52%	*Enterobacter*	10.19%	Firmicutes	21.29%	*Klebsiella*	13.26%	Proteobacteria	23.94%	*Lactobacillus*	14.17%
Firmicutes	11.51%	*Pseudocitrobacter*	4.60%	Tenericutes	5.11%	*Lactococcus*	10.21%	Actinobacteria	6.32%	*Weissella*	9.43%
Tenericutes	1.27%	*Lactobacillus*	4.07%	Cyanobacteria	2.00%	*Rickettsia*	5.38%	Bacteroidetes	3.69%	*Hafnia-Obesum bacterium*	3.78%

However, bacterial community compositions at the genus level differed widely among insect species and feeding habits. Insect taxonomy and feeding habits could be the driving forces for the observed differences, bacterial community compositions were similar in the same family taxa with the same feeding habits. Further analysis revealed that the most abundance top 4 bacteria genera in omnivorous insects were *Lactococcus*, *Lactobacillus*, *Weissella* and *Hafnia-Obesumbacterium*, whereas in carnivorous insects were *Serratia*, *Klebsiella*, *Lactococcus* and *Rickettsia*, and herbivorous insects were unclassified members, *Enterobacter*, *Pseudocitrobacter* and *Lactobacillus*. *Wolbachia* was the dominant genus in *A*. *cinerea* and accounted for 49.46%, but was unobserved in all Ensifera insects but one sample of *H*. *japonicas* (Fig 4B, Table 3). The most dominant genera of omnivorous insects belong to the phylum Firmicutes, whereas the dominant genera of herbivores and carnivores insects mostly belong to the phylum Proteobacteria. A few taxa that occurred without a top 30-abundance are included in ‘Other’.

**Fig 5:**
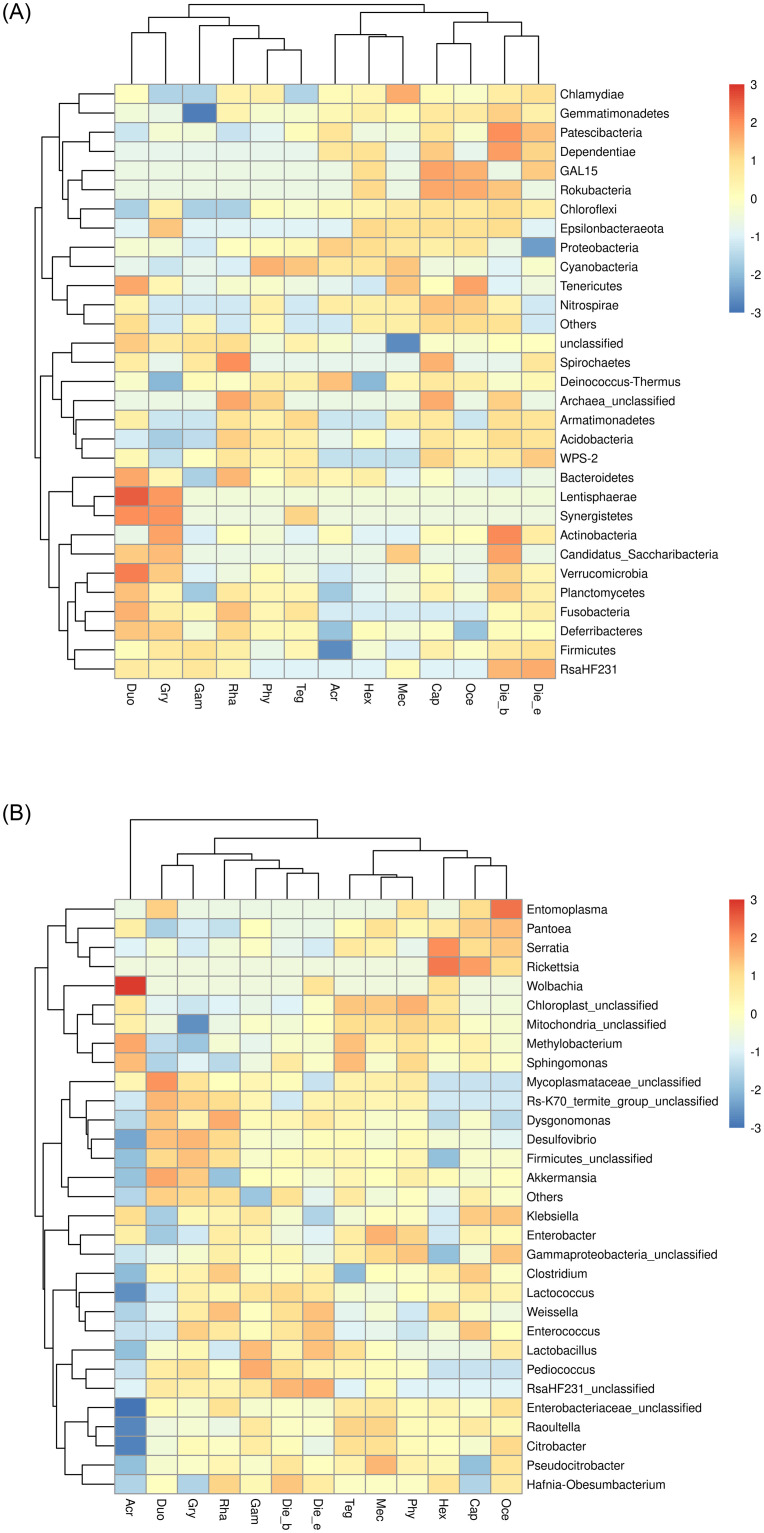
A clustered heatmap using the corresponding dendrogram illustrating the top 30 mean abundances of the bacterial community taxa assigned to phyla (A) and genera (B) level. The color scale of higher (red) and lower (blue) show the relative abundances of bacterial communities.

The top 30 relative abundances of phyla and genera were considered for the heat map cluster analysis. The prevalence heat map revealed that the presence of gut bacterial communities at phylum level in each sample was more clustered according to insect taxonomy, such as the *M*. *niponensis* and *H*. *japonicus* belonged to Tettiidae, *O*. *emeiensis* and *C*. *spinose* belonged to Gryllacrididae, and *D*. *excavate* and *D*. *Beybienkoi* belonged to Rhaphidophoridae (Fig 5A). Furthermore, with the improvement of taxonomical classification of gut bacterial communities at the genus level, it was found that samples were clustered closely based on feeding habits, such as carnivorous Ensifera of *O*. *emeiensis*, *C*. *spinose* and *H*. *japonicas*, herbivorous Ensifera of *M*. *niponensis*, *T*. *novaehollandiae viridinotata*. *P*. *sinicus*. *H*. *japonicas* and *G*. *gratiosa* exhibited different feeding habits were clustered in different branches compared with other samples belonged to Tettigoniidae. Specifically, *A*. *cinerea* belonged to the suborder of Caelifera was clustered separately from others belonged to the suborder of Ensifera (Fig 5B).

### Bacterial diversity as a biomarker for samples with different feeding habits

**Fig 6:**
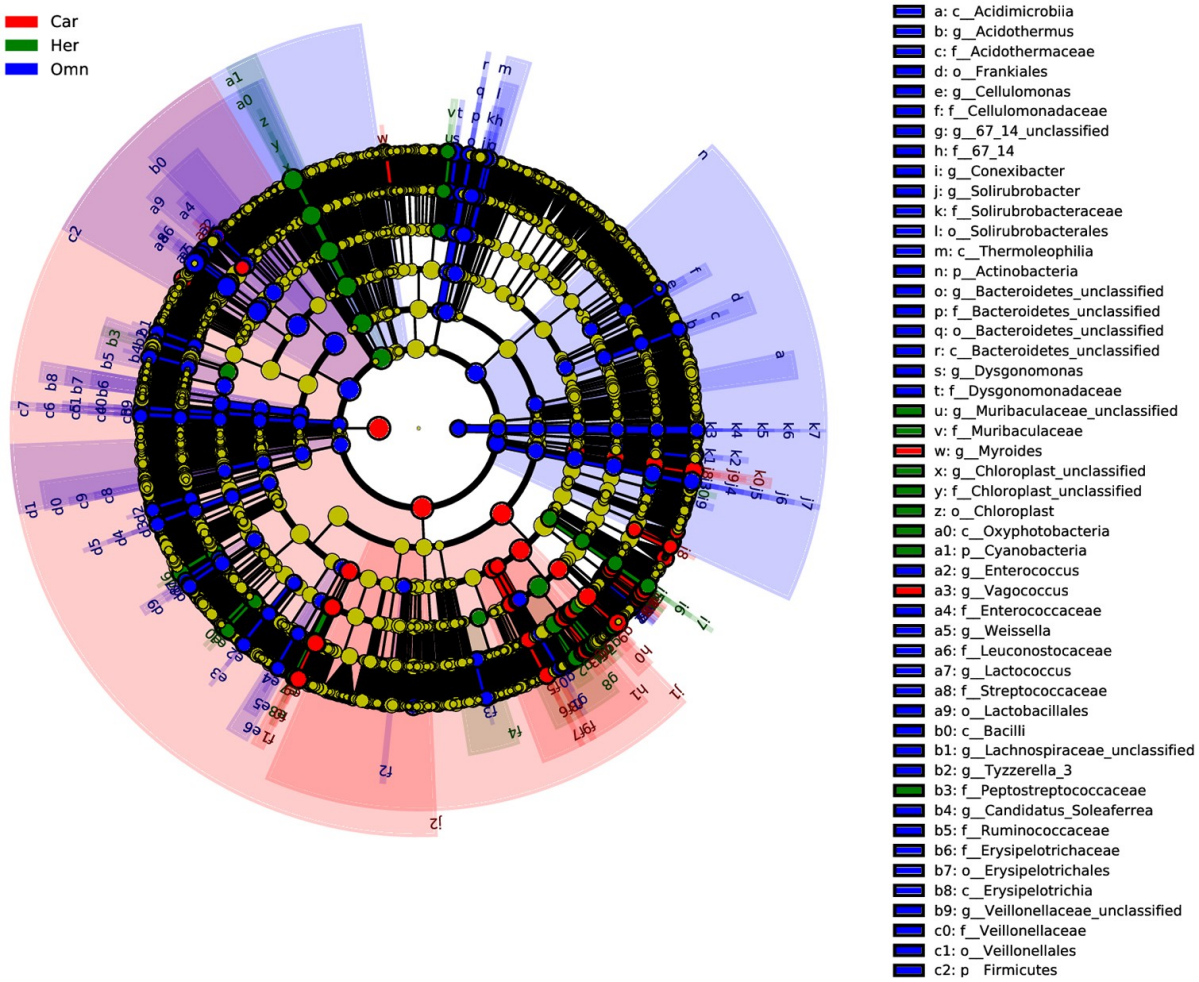
LEfSe analysis: Biomarkers associated with three feeding type. Car: Carnivores; Her: Herbivores; Omn: Omnivores.

Linear discriminant effect size (LEfSe) analysis was conducted for biomarker discovery associated with the three groups of herbivorous, carnivorous and omnivorous Ensifera (LDA scores 3.0) [37,38]. Overall, the Firmicutes, Actinobacteria, RsaHF231, Planctomycetes and Fusobacteria were identified as potential biomarkers for omnivores, Cyanobacteria for herbivores and Proteobacteria for carnivores on phylum level (Fig 6 and S3 Fig). While most bacteria as biomarkers for specific dietary characteristics of insects showed high abundance, some were deemed the core taxa. Similar results were found at the genus level; bacterial taxa in the top 4-abundance were identified as part of potential biomarkers, such as *Lactococcus* for omnivores, Chloroplast_unclassified, *Enterobacter* and *Pseudocitrobacter* for herbivores, and *Serratia*, *Klebsiella*, and *Rickettsia* for carnivores (S4 Fig). Moreover, some bacterial genera with low abundance were also found to be potential biomarkers, indicating that both high and less abundant bacterial taxa perform important roles in shaping insect-dietary pattern [39].

### Common and unique bacteria of samples with different characters

According to gut bacterial communities of insects with three feeding habits, 11 bacterial phyla such as Proteobacteria, Firmicutes, Cyanobacteria, Actinobacteria, Tenericutes, Bacteroidetes (account for > 1%), Verrucomicrobia, Planctomycetes, Acidobacteria, Patescibacteria and Gemmatimonadetes (account for < 1%) from more to less were overlapped by all samples, whereas, none of these insect samples with the same feeding habits harboured unique bacteria on phyla level. However, according to insect taxonomy, the samples from the family Gryllacrididae harboured unique bacteria of Zixibacteria (account for < 1%) and Omnitrophicaeota (account for < 1%) on phyla level [26].

**Fig 7:**
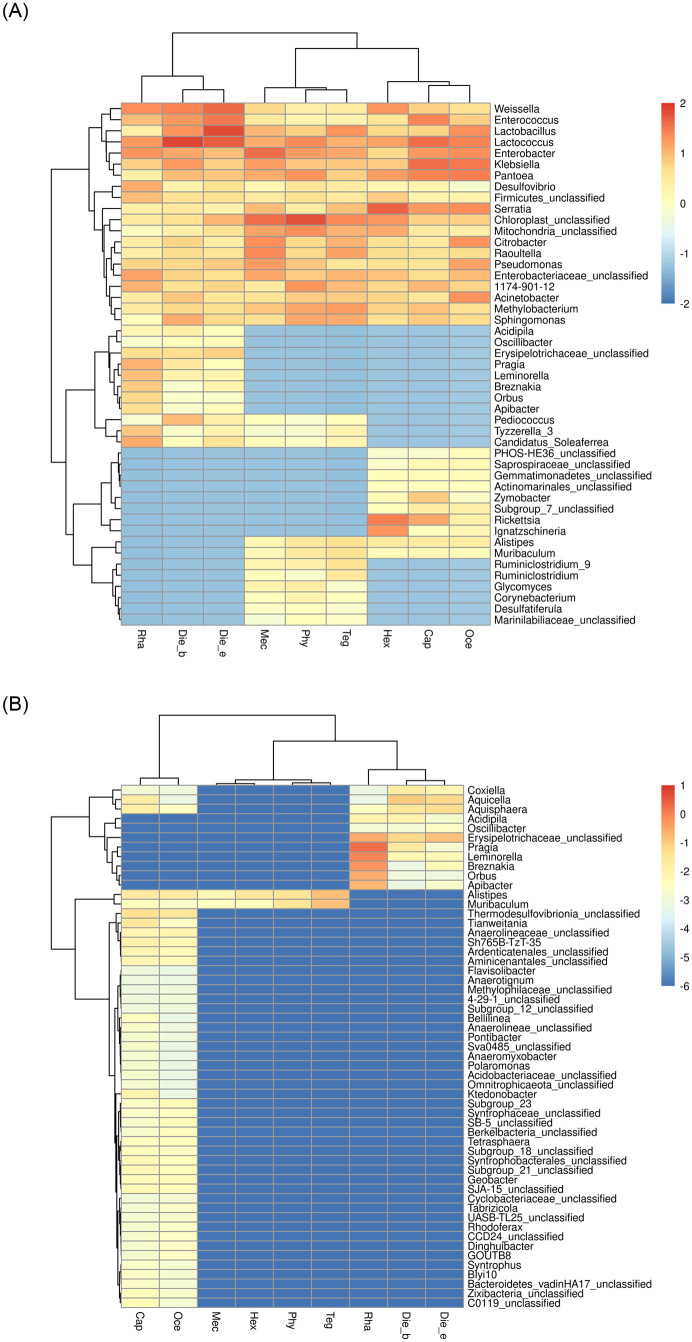
Heat map illustrating the common and endemic bacteria community abundances according to insects feeding habits (A) and insects taxonomy (B) at the genus level. The color scale of higher (red) and zero (blue) show the relative abundances of bacterial communities.

Conversely, gut bacterial communities of insects at the genus level with three feeding habits, 52 bacterial genera were overlapped by all samples, and the top 20 bacterial genera contributed 90% abundance of bacterial communities. Herbivores overlapped the number of bacterial genera were 3 and 2 of omnivores and carnivores, respectively. However, omnivores and carnivores overlapped no bacterial communities. Meanwhile, each feeding habit samples had unique bacteria at the genus level, among which, *Pragia* and *Leminorella* for omnivores, *Rickettsia* and *Ignatzschineria* for carnivores were potential biomarkers. However, no bacterial genus served as biomarkers was found in unique bacteria of herbivores (Fig 7A). Furthermore, the proportions of unique bacterial communities were < 1%, except for *Rickettsia* and *Ignatzschineria* as biomarkers for carnivores.

From the perspective of host taxonomy, the common bacterial genera of 9 insect species at the gut bacterial genus level were the same as those mentioned above. The overlapped bacterial genera were 2 of the samples from Tettigoniidae and Gryllacrididae, and 3 of the samples from Gryllacrididae and Rhaphidophoridae, but no common bacterial genus in samples from Tettigoniidae and Rhaphidophoridae was found. Meanwhile, in the single feeding habits of insect samples belonging to the same family, they had specific bacterial genera, such as Gryllacrididae and Rhaphidophoridae possessed 42 and 8 endemic bacterial genera, respectively. However, no unique genus observed in Tettigoniidae with diverse feeding habits (Fig 7B).

### Assess dietary pattern for insects with contrasting feeding habits

According to the information about the unique gut bacteria and observation of insect taxonomy, although samples from same family taxa with single feeding habits had unique bacteria, the Tettigoniidae with multiple feeding habits did not harbour unique bacteria under this classification. However, from the dietary point of view, samples from different taxa with the same feeding habits had unique bacteria. Therefore, the information of biomarker, unique and common bacteria of different feeding habits of Ensifera were used to analyse and predict samples with contrasting feeding habits, such as *D*. *dendrophilus*, *G*. *orientalis* and *G*. *gratiosa*.

According to alpha diversity analysis, the indices of *D*. *dendrophilus* and *G*. *orientalis* showed higher diversity than that of herbivorous and carnivorous Ensifera, while they were close to omnivores Ensifera. However, the indices of *G*. *gratiosa* indicated low diversity. Meanwhile, the results of beta diversity showed that *D*. *dendrophilus*, *G*. *orientalis* and *G*. *gratiosa* were all clustered with omnivorous Ensifera, as the same results of clustered heat map of the bacterial community taxa assigned to the genus level.

Ensifera samples with contrasting feeding habits were analysed using the dominant bacterial community composition from the Ensifera samples with definite feeding habits. The results showed that the gut bacterial community composition of *D*. *dendrophilus* corresponded to those of carnivores, while *G*. *orientalis* and *G*. *gratiosa* conformed to those of omnivores at the phylum level. The results of gut bacterial community composition at the genus level showed that Firmicutes were the dominated bacterial community of *G*. *orientalis* and *G*. *gratiosa* that was more similar to the omnivores. The gut bacterial community structure of *D*. *dendrophilus* at the genus level was complicated to infer about their feeding habits (Tables 3 and 4). However, compared with the predominant phyla of herbivores, *D*. *dendrophilus* did not harbour much abundance of Cyanobacteria (Cyanobacteria abundance was < 0.2% in all specimens) which as a biomarker contributed 31.52% of the bacterial communities of herbivores, indicated that *D*. *dendrophilus* was not herbivores. Similarly, *G*. *orientalis* and *G*. *gratiosa* were not herbivores either.

**Table 4:** Assess dietary pattern using dominant bacterial community composition.

Species		*Duolandrevus dendrophilus*		*Gryllotalpa orientalis*		*Gampsocleis gratiosa*	
Taxonomy on bacteria		Phlya	Genera	Phlya	Genera	Phlya	Genera
**Identification of bacteria**	**Top1**	Proteobacteria	Mycoplasmataceae_unclassified	Firmicutes	Firmicutes_unclassified	Firmicutes	*Lactobacillus*
	**Top2**	Firmicutes	*Akkermansia*	Proteobacteria	*Desulfovibrio*	Proteobacteria	*Lactococcus*
	**Top3**	Tenericutes	Rs-K70_termite_group_unclassified	Actinobacteria	*Lactococcus*	<5%	*Pediococcus*
	**Top4**	Verrucomicrobia	*Desulfovibrio*	<5%	*Enterococcus*		*Klebsiella*
**Predictions of feeding habits**		Carnivores	Unpredictable	Omnivores	Omnivores	Omnivores	Omnivores

Insects with contrasting feeding habits were analysed using unique bacteria, and the results showed that compared with 6 and 8 unique bacterial species of herbivores and omnivores, respectively (Fig 7A and Table 5), *D*. *dendrophilus*, *G*. *orientalis* and *G*. *gratiosa* harboured several unique bacteria of both herbivorous and omnivorous insects. Furthermore, *D*. *dendrophilus* and *G*. *gratiosa* contained *Pragia*–a biomarker for omnivorous insects, while *D*. *dendrophilus*, *G*. *orientalis* and *G*. *gratiosa* contained no unique bacteria of carnivorous insects (except one specimen of *G*. *orientalis* had 0.00782%), indicating that these samples were not carnivores.

**Table 5:** Assess dietary pattern using unique bacteria of insects with different feeding habits.

Unique bacteria	Samples harboured unique bacteria of insects with different feeding habits		
**Herbivores**	***Duolandrevus dendrophilus***	***Gryllotalpa orientalis***	***Gampsocleis gratiosa***
*Desulfatiferula* Marinilabiliaceae_unclassified *Ruminiclostridium_9* *Ruminiclostridium* *Glycomyces* *Corynebacterium*	*Desulfatiferula* Marinilabiliaceae_unclassified	*Desulfatiferula* Marinilabiliaceae_unclassified *Ruminiclostridium* *Glycomyces* *Corynebacterium*	*Desulfatiferula* *Ruminiclostridium_9* *Corynebacterium*
**Omnivores**	***Duolandrevus dendrophilus***	***Gryllotalpa orientalis***	***Gampsocleis gratiosa***
*Pragia* *Leminorella* *Breznakia* Erysipelotrichaceae_unclassified *Orbus* *Apibacter* *Acidipila* *Oscillibacter*	*Pragia* *Breznakia* Erysipelotrichaceae_unclassified *Acidipila*	*Breznakia*	*Pragia* Erysipelotrichaceae_unclassified
**Carnivores**	***Duolandrevus dendrophilus***	***Gryllotalpa orientalis***	***Gampsocleis gratiosa***
*Rickettsia* *Ignatzschineria* *Zymobacter* Subgroup_7_unclassified Gemmatimonadetes_unclassified Saprospiraceae_unclassified Actinomarinales_unclassified PHOS-HE36_unclassified	None	None	None
**Prediction of feeding habits**	Not carnivores	Not carnivores	Not carnivores

In summary, we inferred that the feeding habits of *D*. *dendrophilus*, *G*. *orientalis* and *G*. *gratiosa* were omnivores.

## Discussion

### Important factors affecting the structure of gut bacterial community

The host diet and taxonomy were the two most important factors shaping their gut microbiome [11,40]. While some studies have revealed that diet played an overriding guidance role in microbiome structure, others suggested that common ancestry dominates the influence in determining the microbiome structure [41,42], and similar studies have not been reported in Ensifera. In this study, the gut bacterial communities were characterized across 13 wild species exhibited different feeding habits properties. Among them, 41 bacterial phyla represented 120 classes, 272 orders, 504 families and 1284 genera were obtained. Similar to the gut bacterial community of those reported in arthropods and mammals, Ensifera harboured bacterial taxa of Proteobacteria, Firmicutes, Cyanobacteria, Actinobacteria and Tenericutes [43–46]. However, the bacterial community structure was not only different between the two orders, but also different within the order. These difference mainly relation to dietary factors, such as Proteobacteria was the dominant phylum in carnivores and herbivores, and Firmicutes was the dominant phyla in omnivores. The second dominant phylum in herbivores was Cyanobacteria in contrast to previous studies as Proteobacteria and Firmicutes, which were the predominant bacterial phyla in all insect gut samples [10,11,44,47]. Conversely, Cyanobacteria were very rare and had a low abundance in some insects [48,49]. However, it had been reported to be the most substantial bacteria in vertebrate animals, especially in herbivorous fishes with a rather higher abundance of more than 34% [50], While, Cyanobacteria were with a low abundance in omnivorous and carnivorous Ensifera, which was consistent with the results not detected in carnivorous fish. This indicated that the feeding habits played a key role in the abundance of Cyanobacteria. Similarly, this characteristic of herbivorous insects also played an important role in the prediction of insect feeding habits. These prokaryotes had the capabilities for nitrogen fixation and oxygen production, and were identified as potential biomarkers for herbivorous Ensifera.

The gut bacterial community structures of Ensifera with three feeding types were significantly different at the genus level. The bacteria of omnivores that overlapped with herbivores and carnivores were *Lactobacillus* and *Lactococcus* in the top 4 abundance genera, respectively. This was consistency in the analysed results of LEfSe that the remaining top 4 abundance genera were biomarkers for herbivores and carnivores. Furthermore, the top 4 abundance genera of the three feeding types of Ensifera belonged to Proteobacteria and Firmicutes. Previous studies have shown that the *Pantoea* genus was responsible for plant cell-wall polymers break down [51]. However, in this study, the *Pantoea* genus has a higher abundance in carnivores than others, also was biomarker for carnivores, indicated that *Pantoea* played a certain role in degrading carnivorous foods.

It is worth noting that *Wolbachia* represented the most prevalent endosymbiotic bacterial group, affecting around 40% of arthropod species [52,53]. Surprisingly, *Wolbachia* was the only dominant genus in *A*.*cinerea* and accounted for 49.46%, but was unobserved in all Ensifera samples. Although there were significant interactions between *Wolbachia* and host from parasitic to mutualistic, the most frequently noted parasitic effects were reproductive manipulation by male-killing, feminisation, sperm–egg incompatibility, cytoplasmic incompatibility and parthenogenesis [54,55]. Therefore, we hypothesised that *Wolbachia* could be used as a biological control for Ensifera pests [56].

Alpha diversity analysis indicated that the gut bacterial diversity of Ensifera appeared to be lower in herbivores than omnivores, and comparable to that of carnivores. However, dissimilar with mammal, of which bacterial diversity was lowest in carnivores, intermediate in omnivores and highest in herbivores [40]. Meanwhile, omnivorous Ensifera harboured the highest feature number among all samples, which also confirmed that omnivorous Ensifera has the highest gut bacterial community diversity. Additionally, based on PCA and PCoA analysis, more significant difference in the gut bacterial community were found in Ensifera. Firstly, *A*. *cinerea* belonged to Caelifera suborder clustered separately far away from the insect samples belonged to Ensifera suborder. Secondly, gut bacterial communities among insects from Ensifera clustered by feeding habits and taxonomic status, both. However, feeding habits may contributed more than taxonomic status as the driving forces for the observed bacterial community differences. Finally, this differed from some previous studies [42] that confirming diet was not the primary driver of the bacterial community structure in cockroach gut [20]; also regardless of differences in environment and diet, host genetics were determinants of shaping the gut community structure in crickets [18], and sampling location predominantly shaped the bacterial community composition of dragonfly [15]. Previous study had pointed that gut symbionts with critical roles in nutrient provisioning or digestion could positively influence development of the host. These processes were mediated by contact-dependent interactions of bacteria with the gut epithelium. The evolution of gut cells included stem cell proliferation and epithelial cell renewal were crucial for host to adapt to the changes of gut environment [2]. In our study, feeding habits driving forces for gut bacterial community, changes in feeding habits will lead to changes in gut microbiota, feeding habits and gut microbiota played an important role in the process of gut evolution.

Diversification patterns of Ensifera showed that samples from the family of Tettigoniidae, Rhaphidophoridae and Gryllacrididae belonged to the same branch in the phylogenetic tree representing the infraorder of Tettigoniidea, while the samples from the families of Gryllidae and Gryllotalpidae belonged to the infraorder of Gryllidea [57]. Meanwhile, *M*. *niponensis*, *P*. *sinicus* and *T*. *novaehollandiae viridinotata* belonged to a subfamily of Phaneropteridae, which were the three samples with the closest affinity [58]. The results of the relationship between the gut bacterial communities and the taxonomic status of all samples indicated that the gut bacterial communities at phyla and genus levels were not highly correlated with the taxonomic status of insect hosts. However, we could still find a certain pattern at the genus level, that was, the higher taxonomic level of host, the higher correlation between the similarity of gut bacterial community and its taxonomic status. The lower taxonomic level of host, the lower correlation between the similarity of gut bacterial community and its taxonomic status. The results indicated that feeding habits and taxonomic status jointly affected the gut bacterial community composition of the samples from Orthoptera, however, when taxonomy category below the suborder level, the effect of feeding habits dominates. Unfortunately, it was difficult to find a pattern of gut bacterial community in Tettigonidae–the family with a complex feeding habit.

### The characteristics of the gut bacterial communities were used to evaluate insect feeding habits

The diversity profile of gut microbes across the members of the Ensifera were influenced by the nutritional provisioning by the gut symbionts and the variations in gut anatomy/redox conditions inside the gut. In the function of nutritional provisioning, part of the role of gut microbes is to help digest food, and this mechanism is done by enzymes secreted by the microbes [59,60]. Enzyme catalysis was specific, and different foods require specific enzymes for digestion and absorption by insects, and might adapt to dietary changes through the induction of enzyme production [23]. Different dietary characteristics may ultimately correspond to different gut microbial compositions [61,62]. In this study, we considered the correlation between feeding habits and the gut bacterial community of Ensifera to explore the dietary characteristics of the sample with contrasting feeding habits from the perspective of gut bacterial community. Previous studies have shown that feeding habits could be determined using direct observation for some diurnal insects [63] and DNA sequence analysis was used for identifying gut contents for some insects that have difficulty in directly observing foraging behaviour [64–66]. Here, the bacterial community structure in herbivorous, omnivorous and carnivorous Ensifera was focused on, of which different gut bacterial community composition characteristics lacking unique bacteria, even with low abundance was found, and also common bacteria in the two or three feeding habits of Ensifera was found. Furthermore, feeding behaviour of *D*. *dendrophilus*, *G*. *orientalis* and *G*. *gratiosa* were evaluated by gut bacteria with these characteristics. Additionally, although the feeding habits of the three insects were contrast, scientists preferred to classify them as omnivorous. The prediction results of gut bacterial community structure basically conformed to common sense, indicated that the characteristics of gut microbes could be used to correlate with their host-feeding habits. In the follow-up study, more gut microbial information of samples will be required to systematically establish this evaluation method.

## Conclusion

The results of Ensifera gut bacterial composition and diversity demonstrated that both Proteobacteria (45.66%) and Firmicutes (34.25%) were the predominant bacterial phyla. However, feeding habits and taxonomic status jointly affected the gut bacterial community composition of the samples from Orthoptera. When taxonomy category below the suborder level, the effect of feeding habits dominates. Among them, omnivorous Ensifera harboured the highest diversity of gut bacteria than herbivores and carnivores. Moreover, common and unique bacteria with biomarkers were found in Ensifera with different feeding habits. These characteristic bacterial communities could be used to predict the contrastic feeding habits of insects belonging to Ensifera.

## Supporting information

S1 Fig Venn diagram of the gut bacterial communities features in 13 insect samples. (TIFF)

S2 Fig Box plots of (A) Observed_species, (B) Chao1, (C) Shannon, (D) Simpson values for comparison of bacterial diversity in insect samples. (TIFF)

S3 Fig LEfSe analysis of samples with three feeding habits on gut bacteria phylum level (LDA>3.0). Car: Carnivores; Her: Herbivores; Omn: Omnivores.
(TIFF)

S4 Fig LEfSe analysis of samples with three feeding habits on gut bacteria genus level (LDA>3.0). Car: Carnivores; Her: Herbivores; Omn: Omnivores.
(TIFF)
