# Supplementary figures and images for: Gut bacterial communities across 12 Ensifera (Orthoptera) at different feeding habits and its prediction for the insect with contrasting feeding habits
Source: PLoS One. 2021 Apr 26;16(4):e0250675. doi: 10.1371/journal.pone.0250675 (PMC8075264; doi:10.1371/journal.pone.0250675)

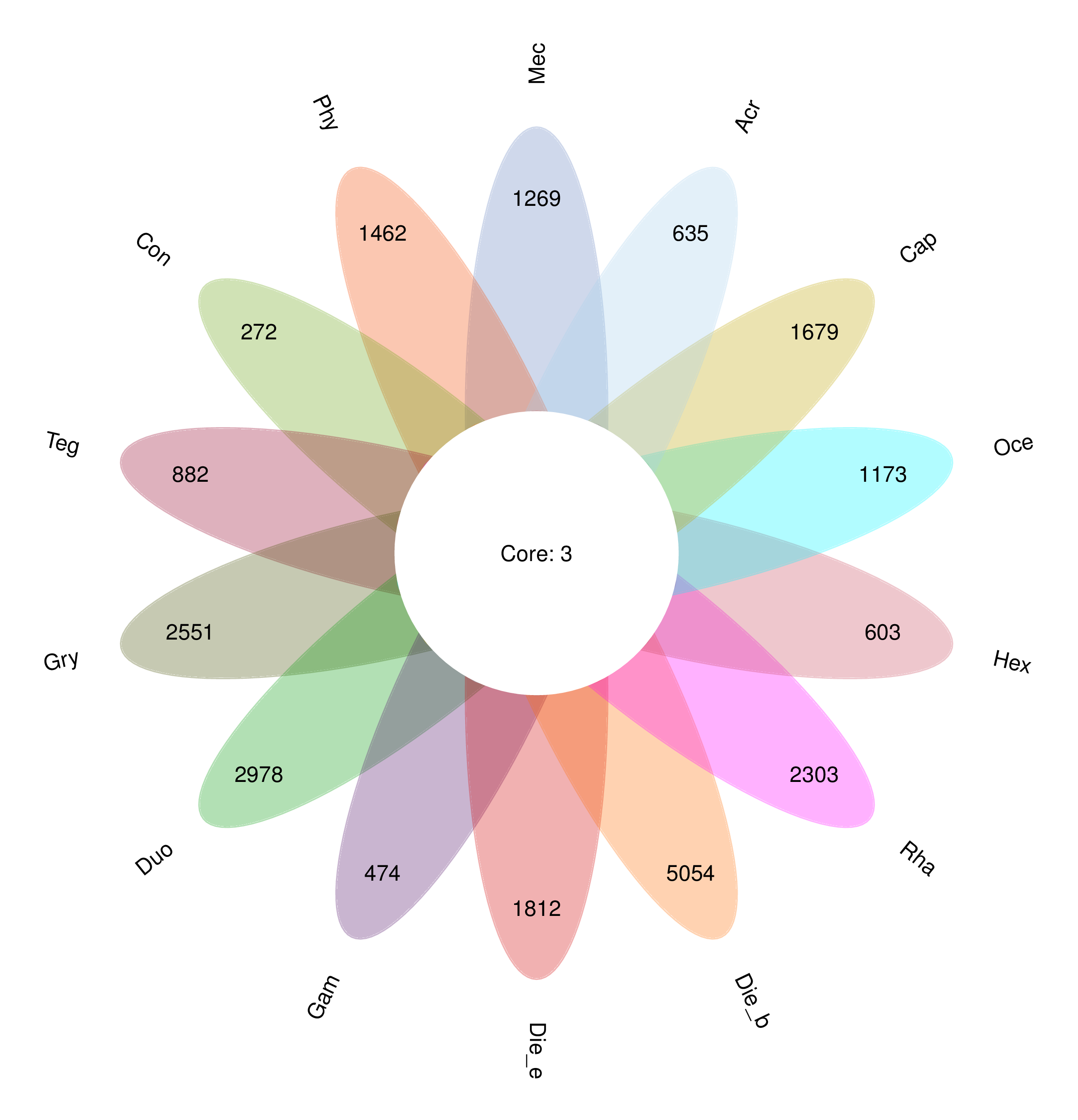

Supplement: S1 Fig — (TIFF) [file pone.0250675.s001.tiff]

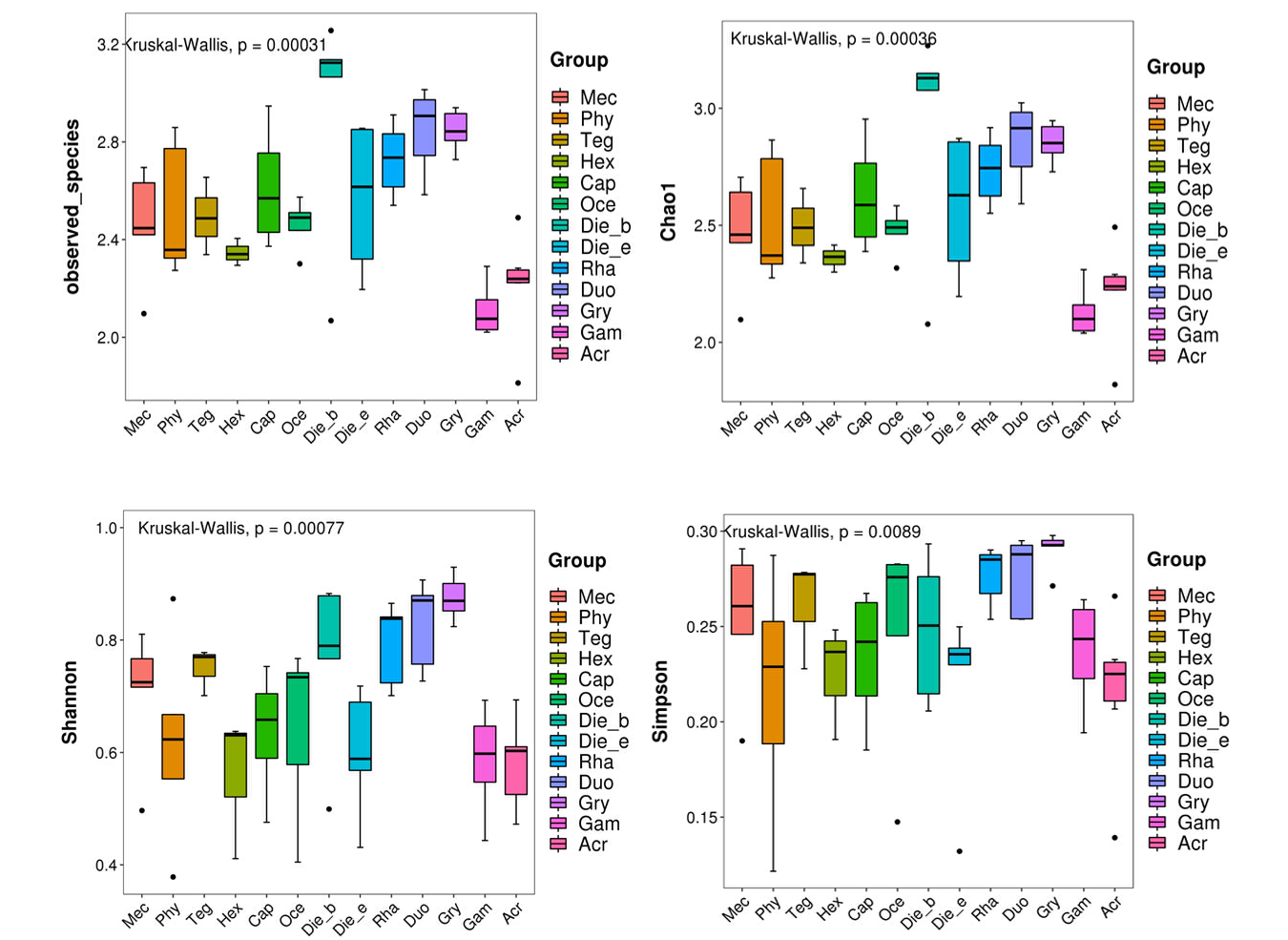

Supplement: S2 Fig — (TIFF) [file pone.0250675.s002.tiff]

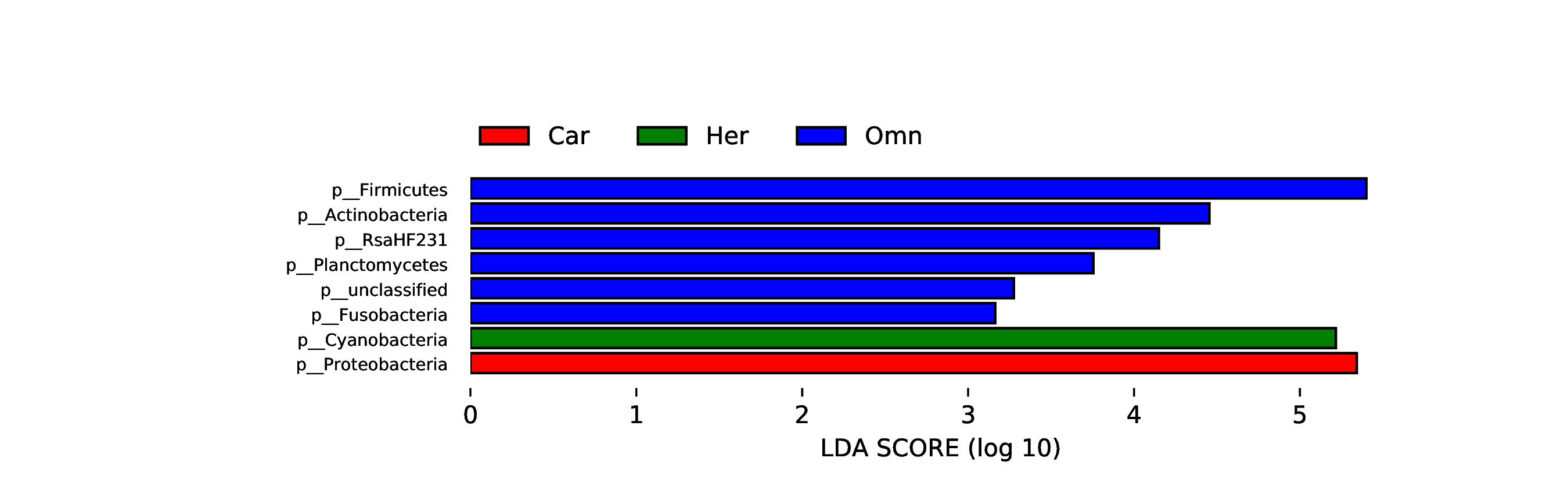

Supplement: S3 Fig — Car: Carnivores; Her: Herbivores; Omn: Omnivores. (TIFF) [file pone.0250675.s003.tiff]

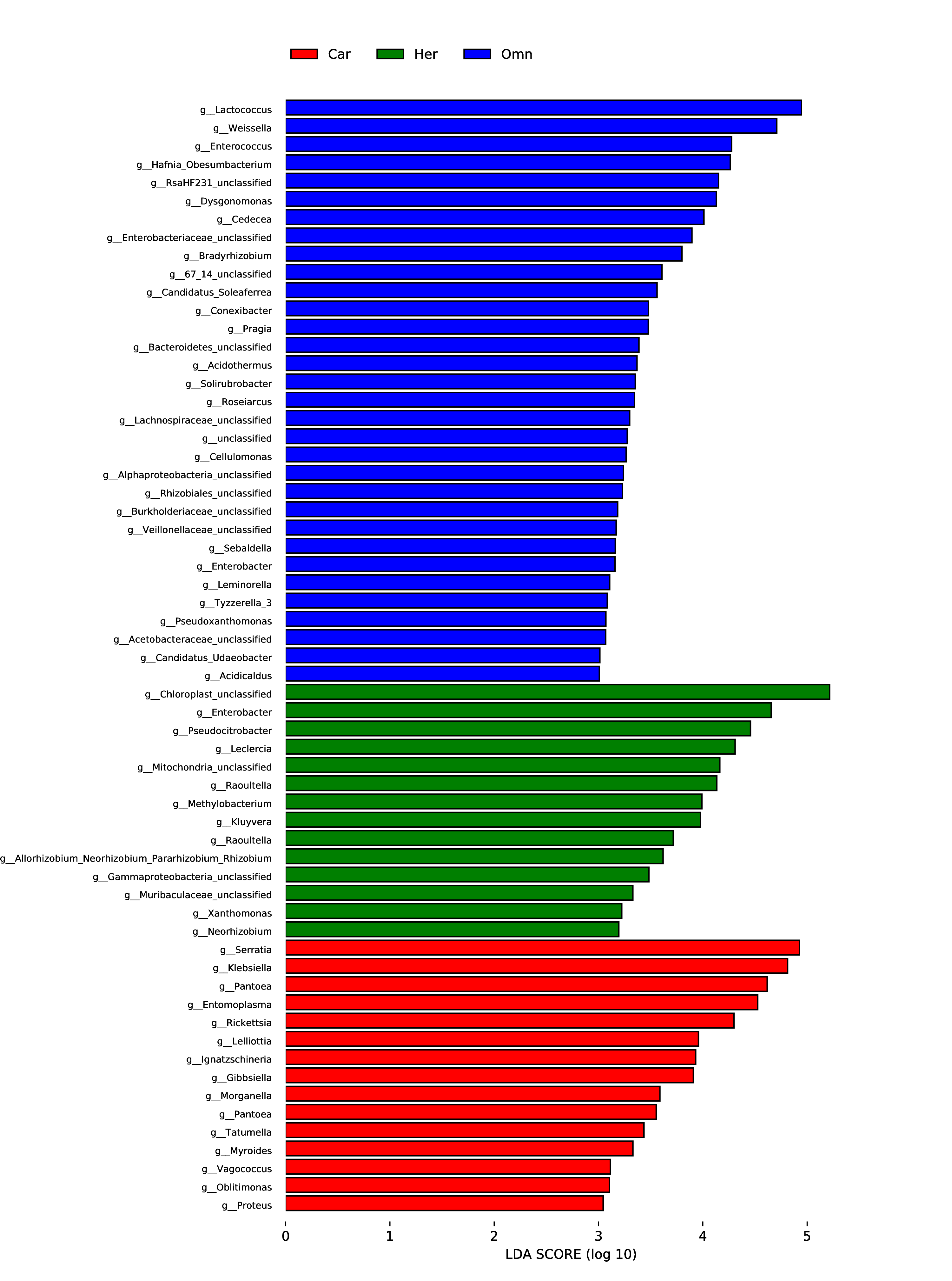

Supplement: S4 Fig — Car: Carnivores; Her: Herbivores; Omn: Omnivores. (TIFF) [file pone.0250675.s004.tiff]
